# Terpenes as bacterial efflux pump inhibitors: A systematic review

**DOI:** 10.3389/fphar.2022.953982

**Published:** 2022-10-13

**Authors:** Kaio Jefté Santos De Oliveira Dias, Gustavo Marinho Miranda, Jonatas Reis Bessa, Ana Carolina Justino De Araújo, Priscilla Ramos Freitas, Ray Silva De Almeida, Cícera Laura Roque Paulo, José Bezerra De Araújo Neto, Henrique D. M. Coutinho, Jaime Ribeiro-Filho

**Affiliations:** ^1^ Laboratory of Genetics and Translational Hematology, Oswaldo Cruz Foundation (FIOCRUZ), Salvador, Bahia, Brazil; ^2^ Institute of Psychology, Federal University of Bahia (UFBA), Salvador, Bahia, Brazil; ^3^ Laboratory of Microbiology and Molecular Biology, Department of Biological Sciences, Regional University of Cariri, Crato, Ceará, Brazil; ^4^ Fiocruz Ceará, Oswaldo Cruz Foundation (FIOCRUZ), Eusébio, Ceará, Brazil

**Keywords:** terpenes, efflux pumps, antibiotic resistance, natural products, systematic review

## Abstract

Managing antibiotic resistance is a significant challenge in modern pharmacotherapy. While molecular analyses have identified efflux pump expression as an essential mechanism underlying multidrug resistance, the targeted drug development has occurred slower. Thus, considering the verification that terpenes can enhance the activity of antibiotics against resistant bacteria, the present study gathered evidence pointing to these natural compounds as bacterial efflux pump inhibitors. A systematic search for manuscripts published between January 2007 and January 2022 was carried out using the Preferred Reporting Items for Systematic Reviews and Meta-Analyses (PRISMA) protocol and the following search terms: “Terpene”; AND “Efflux pump”; and “Bacteria.” From a total of 101 articles found in the initial search, 41 were included in this review. Seventy-five different terpenes, 63 bacterial strains, and 22 different efflux pumps were reported, with carvacrol, *Staphylococcus aureus* SA-1199B, and NorA appearing most frequently mentioned terpene, bacterial strain, and efflux pump (EP), respectively. The Chi-Squared analysis indicated that terpenes are significantly effective EP inhibitors in Gram-positive and Gram-negative strains, with the inhibitory frequency significantly higher in Gram-positive strains. The results of the present review suggest that terpenes are significant efflux pump inhibitors and, as such, can be used in drug development targeting the combat of antibacterial resistance.

## Introduction

The introduction of antibiotics in the medical scenario in the 1940s dramatically reduced mortality rates due to bacterial infections. It revolutionized the treatment of diseases requiring surgical procedures, significantly improving life expectancy worldwide (Habboush and Guzman, 2022). On the other hand, the increased access without proper sanitary control has resulted in irrational antibiotic use, significantly contributing to the development of antibiotic resistance (Laxminarayan et al., 2016). Epidemiological studies have revealed that the number of deaths yearly from antibiotic resistance worldwide is approximately 700,000. Additionally, it is estimated that this number could rise to 10 million by 2050 if significant improvements in antibiotic drug development are not achieved (Willyard, 2017). Consequently, the management of antimicrobial resistance, as well as the reduction in the associated morbidity and mortality rates, currently represent a significant challenge in public health care (Reygaert, 2018; Sharma et al., 2020).

Antibiotic resistance can be defined as the process by which bacteria evolve, causing antibiotics to become less effective against infections they were developed to treat (Murray et al., 2022). As a significantly complex phenomenon, antibiotic resistance can arise from and be affected by various factors, among which bacteria-antibiotic interaction, mutation, and transmission rates in the population, are highlighted (Holmes et al., 2016). Accordingly, bacterial resistance may be due to Intrinsic Resistance, Acquired Resistance, Genetic Change, or DNA Transfer (Habboush and Guzman, 2022).

Despite the increasing variety of molecular mechanisms allowing bacteria to overcome the action of an antibiotic, the expression of efflux proteins stands out for its widespread occurrence among resistant pathogens (Munita and Arias, 2016; Chatterjee et al., 2018). The antibacterial resistance mediated by efflux pumps (EPs) consists of the lack of effectiveness of antibiotics due to their inability to reach the molecular target at an inhibitory concentration (Tortora., 2012; Blanco et al., 2016). While some efflux pumps are substrate-specific, others can mediate the active transport of several compounds, contributing to the development of multidrug resistance (Van Bambeke et al., 2000; Piddock., 2006). These proteins are classified according to their composition, energy source, and the number of transmembrane regions (Sun et al., 2014) and can be grouped into five major families: ATP binding cassette (ABC), small multidrug resistance (SMR), multidrug and toxin extrusion (MATE), major facilitator superfamily (MFS), and resistance nodulation cell division (RND). Except for RND, which is found only in Gram-negative bacteria, all others are present in Gram-positive and Gram-negative bacteria (Pathania et al., 2019).

Cell wall biosynthesis inhibitors (CBIs), such as β-lactams and glycopeptide antibiotics, and cell membrane inhibitors, such as polymyxins and daptomycin, stand out as the most extensively used antibiotic classes (Epand et al., 2016; Sarkar et al., 2017). However, an increasing number of bacterial strains have developed resistance against these drugs (Schwarz et al., 2017; Singh et al., 2017). Therefore, drug research and development targeting resistance mechanisms are highly prioritized (Shrivastava et al., 2018). In this context, consistent evidence has demonstrated that natural products represent promising sources of new bioactive compounds (Huang et al., 2021), among which terpenes stand out for their promising antibacterial properties (Swamy et al., 2016; Mahizan et al., 2019).

A large body of research conducted by scientists all over the world, among which our group is included, has demonstrated that terpenes such as safrole, α-pinene, thymol, carvacrol, limonene, and eugenol are intensely active against Multiple Drug Resistance (MDR) strains (Barbieri et al., 2017; Limaverde et al., 2017; Oliveira-Tintino et al., 2018; de Figueiredo et al., 2019; Almeida et al., 2020; Araújo et al., 2020; Freitas et al., 2020; Muniz et al., 2021). In addition, it has been demonstrated that these compounds can enhance the activity of antibiotics, which is partially due to their ability to inhibit the activity of efflux pumps in resistant bacteria (Barbosa et al., 2021).

Therefore, the present systematic review gathered evidence of terpenes as bacterial efflux pump inhibitors, discussing their potential impact on antibiotic resistance-targeted drug development.

## Methods

The present review was conducted from a systematic search of manuscripts in four scientific databases (Pubmed, Medline, Scopus, and EMBASE) using the Preferred Reporting Items for Systematic Reviews and Meta-Analyses (PRISMA) protocol (Page et al., 2021) and the following search terms: “Terpene”; and “Efflux pump”; and “Bacteria.”

This work included original articles published between January 2007 and January 2022 addressing the potential of fully characterized terpenes as potential bacterial efflux pump inhibitors through *in vivo*, *in vitro*, and *in silico* research. Articles randomly found during the theoretical reference search which met the inclusion criteria were also included. The exclusion criteria were the following: 1) studies demonstrating the effects of terpenes as components of essential oils, extracts, or other complex formulations; 2) articles investigating the participation of efflux pumps in the mechanism of resistance to terpenes.

Following these eligibility criteria, the search was conducted by KJSOD and ACJA. Further, JRF and JRC analyzed and filtered the eligible studies based on their abstracts. This procedure was executed using the Rayyan—Intelligent Systematic Review tool (Ouzzani et al., 2016).

**FIGURE 1:**
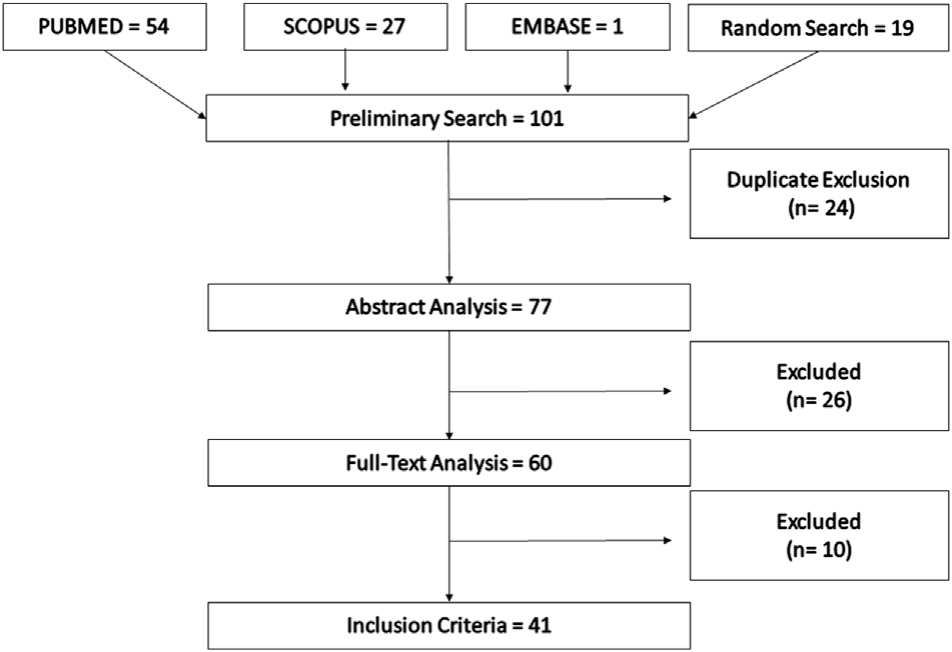
Description of the search and selection of the studies.

The initial search found 101 articles, among which 24 were duplicated. Following the title and abstract analysis, 60 articles were considered eligible for the study. These manuscripts were then double-checked by full-text reading, and 41 articles were included in the final version of this review (Figure 1). The primary data of each study was organized into a table describing the reference, terpene, bacterial strain, efflux pump, and main findings (Table 1).

**TABLE 1:** Summary of the selected studies and their main findings.

Authors	Terpenes	Strain	Efflux Pumps	Results
Almeida et al. (2020)	Safrole	*Staphylococcus aureus* SA-1199B	NorA	Sub-inhibitory concentrations of safrole significantly reduced the MIC of EtBr norfloxacin and ciprofloxacin against the 1199B and K2068 strains of *S. aureus*, suggesting inhibition of the NorA and MepA efflux pumps, which was corroborated by *in silico* analysis
		*Staphylococcus aureus* K2068	MepA	
Barbosa et al. (2021)	Carvacrol	*Staphylococcus aureus* SA-1199	NorA	The results showed that both carvacrol and thymol caused a significant reduction in the MIC of Norfloxacin and EtBr in *S. aureus* strains carrying the NorA efflux pump and docking analysis suggested that these terpenes act as competitive NorA inhibitors
	Thymol	*Staphylococcus aureus* SA-1199B		
Bezerra et al. (2020)	Estragol	*Staphylococcus aureus* RN-4220	MsrA^a^	The association between estragole and EtBr resulted in antagonism, suggesting that the terpene does not act as an EP inhibitor
Cirino et al. (2014)	Carvacrol	*Staphylococcus aureus* SA-1199B	NorA	Both terpenes increased the inhibitory effects of tetracycline against *S. aureus* strains overexpressing the TetK, NorA, and MsrA proteins, suggesting that they may act as EPI
	Thymol	*Staphylococcus aureus* IS-58	TetK	
		*Staphylococcus aureus* RN-4220	MsrA^a^	
Coêlho et al. (2016)	Nerol	*Staphylococcus aureus* SA-1199B	NorA	All these bioactive terpenes, especially nerol, enhanced the activity of norfloxacin against the *S. aureus* strain 1199B (NorA overexpression), which was interpreted as a result of EP inhibition
	3,7-Dimethyl-octanol			
	Estragole			
Cruz et al. (2020)	α-Bisabolol	*Staphylococcus aureus* SA-1199B	NorA	α-Bisabolol reduced the MIC of tetracycline and norfloxacin to clinically relevant values in *S. aureus* strains that overexpressed the NorA and TetK EP. The inclusion complex α-bisabolol β-CD showed lower modulating activity
		*Staphylococcus aureus* IS-58	TetK	
Cuaron et al. (2013)	Terpinen-4-ol	*Staphylococcus aureus* SH1000	RND	The expression of the *mmpL* gene, which encodes proteins of the RND family, was upregulated following the treatment with terpinen-4-ol, providing a potential mechanism of EP- mediated resistance to terpenes by *S. aureus* strain SH 1000
Da Costa et al. (2021)	Estragole	*Staphylococcus aureus* SA-1199B	NorA	The association between estragole and EtBr resulted in synergism against *S. aureus* 1199B and *S. aureus* K2068, suggesting this terpene could inhibit the NorA and MepA proteins in these strains
		*Staphylococcus aureus* K2068	MepA	
Dwivedi et al. (2015)	3-O-acetyl-urs-12-en-28-isopropyl ester (UA4)	*Escherichia coli* KG4	AcrAB-TolC	The UA4 and UA5 (ursolic acid derivatives) reduced the MIC of tetracycline and EtBr. They inhibited the expression of genes of the *RND* and *ABC* families of transporters, such as *acrA, acrB, tolC*, indicating inhibition of EP expression. *In silico* analysis corroborated these findings
	3-Oacetylurs-12- en-28-n-butyl ester (UA-5)		MacB	
			YojI	
Espinoza et al. (2019)	Epicubenol	*Staphylococcus aureus* K2378	NorA	Among the testing compounds, only 15-copaenol and epicubenol inhibited EtBr efflux by the K2378 strain of *S. aureus*
	15-Copaenol			
	15-Copaenal (6OX)			
	15-Copaenyl acetate (6AC)			
Fadli et al. (2014)	Carvacrol	*Escherichia coli* AG100	AcrAB-TolC	Thymol and carvacrol significantly reduced the expression of proteins forming the AcrAB-TolC complex in the *E. coli* AG100 and *E. coli* AG100A strains, indicating an EP inhibitory role by these terpenes
	Thymol	*Escherichia coli AG*100A		
de Figueiredo et al. (2019)	(4R,14S)-4α,14αdihydroxydolasta-1 (15),8-diene	*Staphylococcus aureus* 1199B	NorA	(4S,9R,14S)-4α-acetoxy-9β,14α-dihydroxydolasta-1 (15),7- diene as well as a mixture of (4R,14S)-4α,14αdihydroxydolasta-1 (15),8-diene and (4R,7R,14S)-4α,7α-diacetoxy-14α-hydroxydolasta-1 (15),8-diene significantly reduced the MIC of tetracycline, erythromycin, and norfloxacin against *S. aureus* strains bearing the NorA, TetK, and MsrA proteins (1199B, IS-58, and RN4220, respectively)
	(4R,7R,14S)-4α,7α-diacetoxy-14α-hydroxydolasta-1 (15),8-diene	*Staphylococcus aureus* RN-4220	MsrA^a^	
	(4S,9R,14S)-4α-acetoxy-9β,14α-dihydroxydolasta-1 (15),7- diene	*Staphylococcus aureus* IS-58	TetK	
Freitas et al. (2020)	α-Pinene	*Staphylococcus aureus* RN-4220	MrsA^a^	α-pinene enhanced the activity of tetracycline against the *S. aureus* IS-58 strain but failed in modify the antibacterial effect of erythromycin against the *S. aureus* RN-4220 strain. The authors suggest inhibition of the TetK EP
		*Staphylococcus aureus* IS-58	TetK	
Freitas et al. (2021)	Limonene	*Staphylococcus aureus K2068*	MepA	Limonene association decreased the MIC of EtBr and ciprofloxacin in addition to showing favorable interaction with the active site of MepA *in silico*. The inhibitory activity was confirmed through analysis of EtBr fluorescence emission intensity
Gupta et al. (2016)	16α-hydroxycleroda-3,13 (14) -Z-dien-15,16-olide	*Staphylococcus aureus* MRSA-ST2071	NorA	This diterpene significantly inhibited EtBr efflux and extended the antibiotic effect of fluoroquinolones in addition to inhibiting the expression of *NorA*, *NorB, NorC, MdeA,* and *MepA* genes
			NorB	
			NorC	
			MdeA	
			MepA	
Gupta et al. (2017)	Citral	*Staphylococcus aureus* MRSA-ST2071	Not Reported	Citral showed a potential EP inhibitory capacity, as indicated by the decreased fluorescence due to reduced EtBr extrusion
Jang and Eom. (2019)	α-humulene	*Bacteroides fragilis* WT-ETBF	RND	α-humulene induced transcriptional changes in the *Bacteroides fragilis*, resulting in a reduced expression of the *bmeB1* and *bmeB2* genes, which indicates inhibition of the RND EP expression in *B. fragilis* strains
		*Bacteroides fragilis* rETBF		
		*Bacteroides fragilis* WT-NTBF		
Jin et al. (2010)	Farnesol	*Mycobacterium smegmatis* mc2 155 ATCC 700084	Not reported	Farnesol decreased the MIC of EtBr and rifampicin and increased the accumulation of EtBr in *M. smegmatis*
Kim et al. (2018)	Celastrol	*Stenotrophomonas maltophilia* ATCC 13637	RND	Celastrol significantly inhibited the expression of the *smeYZ* gene in both strains of *S. maltophilia*. Such an effect was associated with the attenuation of biofilm formation, swimming motility, and protease secretion
		*Stenotrophomonas maltophilia* GNU2233		
Kim et al. (2019)	Zerumbone	*Bacteroides fragilis* WT-ETBF	RND	In the WT-ETBF and rETBF strains, zerubone reduced the *bmeB12* gene expression levels. However, in the WT-NTBF, no significant modulation of gene expression was observed
		*Bacteroides fragilis* WT-NTBF		
		*Bacteroides fragilis* rETBF		
Li et al. (2011)	Artesunate	*Escherichia coli* ATCC 35218	AcrAB-TolC	The terpenoid did not show an antibacterial action but reduced the antibiotic MICs when associated with penicillin, cefpiramide, and ampicillin/sulbactam
		*Escherichia coli* AG100A		
Limaverde et al. (2017)	α-terpinene	*Staphylococcus aureus* IS-58	TetK	α-terpinene showed synergistic effects with tetracycline and EtBr, possibly due to an effect on the Tetk efflux pump of *S. aureus* strain IS-58
Lorenzi et al. (2009)	Geraniol	*Enterobacter aerogenes* EAEP289	AcrAB-TolC	The terpene geraniol increased the effectiveness of β -lactam and quinolone antibiotics, decreasing the MICs of ampicillin, penicillin, and norfloxacin against an *E. aerogenes* strain overexpressing the AcrAB-Tolc efflux pump
Mahmoudi et al. (2020)	Menthol	*Acinetobacter baumannii*	AdeABC	Menthol decreased the antibiotic resistance observed against imipenem and ciprofloxacin in *A. baumannii* isolates overexpressing genes *adeA*, *adeB*, and *adeC*, which incodes efflux pumps
Martins et al. (2011)	Uvaol	*Staphylococcus aureus* MRSA COL_OXA_	Not reported	These terpenes significantly increased the amount of fluorescence of EtBr accumulated in the evaluated strains, in addition to decreasing the antibiotic MIC. Among the compounds, uvaol presented the most potent inhibitory activity
	β-Amyrin	*Enterococcus faecalis*		
	oleanolic acid	AG100TET8		
		*Salmonella enterica serotype Enteritidis*		
		*Mycobacterium tuberculosis*		
Miladi et al. (2017) A	Carvacrol	*Staphylococcus aureus* ATCC 25923	Not reported	All these compounds increased the accumulation of EtBr in bacterial cells, indicating efflux pump inhibition. In addition, these compounds increased the effectiveness of antibiotics and reduced the accumulation of biofilm, which confirms their antibacterial activity
	Eugenol			
	p-cymene			
	Thymol			
	γ-terpinene			
Montagu et al. (2016)	Carvacrol	*Acinetobacter baumannii*	Not reported	Both carvacrol and its lipid nanocapsule incorporated formulation presented a synergistic effect with the efflux inhibitor CCCP, indicating interference with the efflux mechanisms of *A. baumannii*
Mouwakeh et al. (2019)	Carvacrol	*Staphylococcus aureus* ATCC 25923	MepA	Carvacrol induced the accumulation of EtBr in both strains of *S. aureus*. Thymoquinone and p-cymene down-regulated and upregulated, respectively, the expression of MepA in the ATCC strain, whereas in the MRSA strain, the expression of these genes was down-regulated by p-cymene alone
	p-cymene	*Staphylococcus aureus* MRSA 272123		
	Thymoquinone			
Muniz et al. (2021)	Eugenol	*Staphylococcus aureus* 1,199	NorA	Eugenol, as well as its natural and synthetic derivatives, enhanced the effectiveness of norfloxacin and reduced the MIC of EtBr against NorA expressing *S. aureus* 1199B strain, which corroborated the favorable interaction between 4-allyl1-2,6-dimethoxyphenol and NorA demonstrated *in silico*
	Allylbenzene	*Staphylococcus aureus* 1199B		
	Estragole			
	Isoeugenol			
	4-allyl-2,6-dimethoxyphenol			
Ojeda -Sana et al. (2012)	Carnosic acid	*Enterococcus faecalis* ATCC 29212	Not reported	Carnosic acid inhibited the uptake/efflux of EtBr, which correlated with the induction of change in the membrane potential gradient in *S. aureus* and *E. faecalis*
		*Staphylococcu s aureus* ATCC 25923		
Oliveira et al. (2021)	α, β-amyrin	*Staphylococcus aureus* 1199B	NorA	α, β-amyrin showed synergistic effects with CCCP against *S. aureus* strains. Also, *in silico* testing demonstrated that this compound has a higher affinity to the MepA and NorA binding sites than standard antibiotics such as ciprofloxacin and norfloxacin
		*Staphylococcus aureus* K2068	MepA	
Ramalhete et al. (2011)	Balsaminol A	*Staphylococcus aureus* COL_OXA_	NorA	None of the tested compounds presented a significant inhibitory activity against the efflux system of the *S. typhimurium* and *E. coli* strains. However, they all promoted the accumulation of EtBr in MRSA Coloxa and *E. faecalis*, indicating that these terpenes, especially balsaminagenin B, act as EPI in Gram-positive strains
	Balsaminol F	*Enterococcus faecalis* ATCC 29212	AcrAB-TolC	
	Balsaminagenin A	*Salmonella enterica* Typhimurium 5408		
	Balsaminagenin B	*Salmonella enterica* Typhimurium 5408CIP		
	Balsaminoside A	*Escherichia coli* AG100		
	Karavilagenin C	*Escherichia coli AG100*_*TET*_ *100*		
Scherf et al. (2020)	Terpinolene	*Staphylococcus aureus* K4100	QacC	The association of terpinolene with EtBr promoted a reduction in the MIC of the EPI, pointing to a potentiating activity by oxacillin, which suggests EP inhibition–mediated synergism
Silveira et al. (2020)	Carvacrol	*Staphyloccus aureus* IS-58	Tetk	No evidence of EP inhibition in the IS-58 strain of *S aureus* was observed following the association of thymol or carvacrol with EtBr
	Thymol			
Smith et al. (2007a)	Ferruginol	*Staphylococcus aureus* XU212	Tetk and MecA	Ferruginol and 5-epipisiferol potentiated the activity of standard antibiotics against these efflux system–expressing strains of *S. aureus*. Moreover, ferruginol inhibited EtBr efflux in the 1199B strain, which may involve inhibition of the NorA efflux pump
	5-epipisiferol	*Staphylococcus aureus* RN4220	MsrA^a^	
		*Staphylococcus aureus* 1199B	NorA	
		*Staphylococcus aureus* MRSA EMRSA-15	MecA	
		*Staphylococcus aureus* EMRSA-16	MecA	
Smith et al. (2007b)	Totarol	*Staphylococcus aureus* XU212	TetK	Totarol potentiated the activity of standard antibiotics against these efflux system–expressing strains of *S. aureus*, in addition to inhibiting the efflux of EtBr in the K3902 strain, possibly due to the inhibition of the NorA efflux pump
		*Staphylococcus aureus* RN-4220	NorA	
		*Staphylococcus aureus* 1199B	MsrA^a^	
		*Staphylococcus aureus* K3092		
Upadhyay et al. (2014)	Pivaloyl Phytol	*Escherichia coli* CA8000	ABC	From a total of 15 phytol derivatives, these five compounds promoted EtBr efflux inhibition. The expression pattern of the MDREC-KG4 transcript was inhibited in the presence of tetracycline
	3,4,5-trimethoxybenzoyl Phytol	*Escherichia coli* DH5a		
	2,3-Dichlorobenzoyl Phytol	*Escherichia coli* MDREC-KG4		
	Cinnamoyl Phytol			
	Aldehyde Phytol			
Vasconcelos et al. (2018)	Carvacrol	*Mycobacterium tuberculosis* H37Rv (ATCCR 27294)	Not reported	The carvacrol *in vitro* treatment resulted in increased EtBr accumulation in *M. Tuberculosis*. Also, the potentiation of the rifampicin activity indicated that the compound has efflux pump inhibitory activity
Wu et al. (2008)	Andrographolide	*Pseudomonas aeruginosa* PAO1	MexAB-OprM	Andrographolide inhibited the expression of the MexB/rpsL efflux pump gene in both wild-type and MexAB-OprB strains of *P*. *aeruginosa*
		*Pseudomonas aeruginosa* MexAB-OprM		
Yuan and Yuk (2019)	Carvacrol	*Escherichia coli O 157:H7*	MarA	The exposition to sublethal concentrations of thymol and carvacrol resulted in a decreased expression of the MDR efflux pump genes *MarA* and *AcrB* in *E. coli* O 157:H7. Additionally, an EtBr accumulation assay observed a significant loss of EP activity
	Thymol		AcrB	
Zhang et al. (2014)	Ginsenoside 20(S)-Rh2	*Staphylococcus aureus 29,213*	NorA	Ginsenoside 20(S)-Rh2 promoted the intracellular accumulation of ciprofloxacin. It inhibited the efflux of pyronin Y in *the S. aureus* 29,213 strain indicating EP inhibition, which was confirmed *in silico* through the interaction between this terpene and the NorA efflux pump

Legends: MIC: minimum inhibitory concentration; EtBr: Ethidium bromide; EP: efflux pump; EPI: efflux pump inhibitory; CD: cyclodextrin; ETBF: bacteroides fragilis; MDR: multidrug resistance; PA β N: Phenylalanine arginine β–naphthylamide; CCCP: Carbonylcyanide-3-chlorophenyl hydrazine; *S. aureus = Staphylococcus aureus; S. typhimurium: Salmonella typhimurium; E. coli*: *Escherichia Coli*; *P. aeruginosa: Pseudomonas aeruginosa; M. tuberculosis: Mycobacterium tuberculosis;* E*. faecalis: Enterococcus faecalis.*

^a^ As reported in the discussion, evidence has indicated that MsrA proteins are not efflux pumps.

Additionally, IBM SPSS Statistics for Windows, Version 24.0 (IBM Corp. Released, 2016), and Jeffrey Amazing Statistic Program—JASP Team. (2022) were used to carry out a descriptive analysis and its plots of the frequency of the following variables: Year of publication, type of terpene, bacterial strain, and type of efflux pump. Finally, a binary logistic regression was computed in which the effect of the efflux pumps inhibition was the dependent variable, and the Gram classification, i.e., Gram-negative and Gram-positive was the independent variable.

## Results and discussion

Several proteins have been targeted in studies evaluating the activity of new antibacterial compounds, among which the following are highlighted: 1) In the SMR family, the Smr/QacC and EmrE proteins, identified in *Staphylococcus aureus* and *Escherichia coli,* respectively; 2) In the MFS family, the NorA and TetK proteins, expressed by *Staphylococcus aureus*; 3) In the RND family, the AcrB protein, found in *Escherichia coli*; 4) In the MATE family, the NorM protein, expressed by *Neisseria meningitidis*, and 5) In the ABC family, the MacB and MsbA efflux proteins, both identified in *Escherichia coli* (Borges-Walmsley et al., 2003).

The search for efflux pump inhibitors has identified a variety of natural products with the potential to be used in antibacterial drug development, among which terpenes, flavonoids, tannins, and alkaloids stand out for their notable pharmacological activity. However, the clinical development of many of these compounds is limited mainly due to their significant toxicity (Stavri et al., 2007; Prasch and Bucar, 2015). Nevertheless, studies conducted by ours and other groups have highlighted the therapeutic potential of terpenes in the context of antibiotic resistance, emphasizing their role as efflux pump inhibitors (Stavri et al., 2007). Additionally, studies have proven that monoterpenes can affect the structure and function of the bacterial membrane by interacting with membrane components, including polysaccharides, fatty acids, phospholipids, and proteins, facilitating the intracellular action of antibiotics (Sikkema et al., 1994).

**FIGURE 2:**
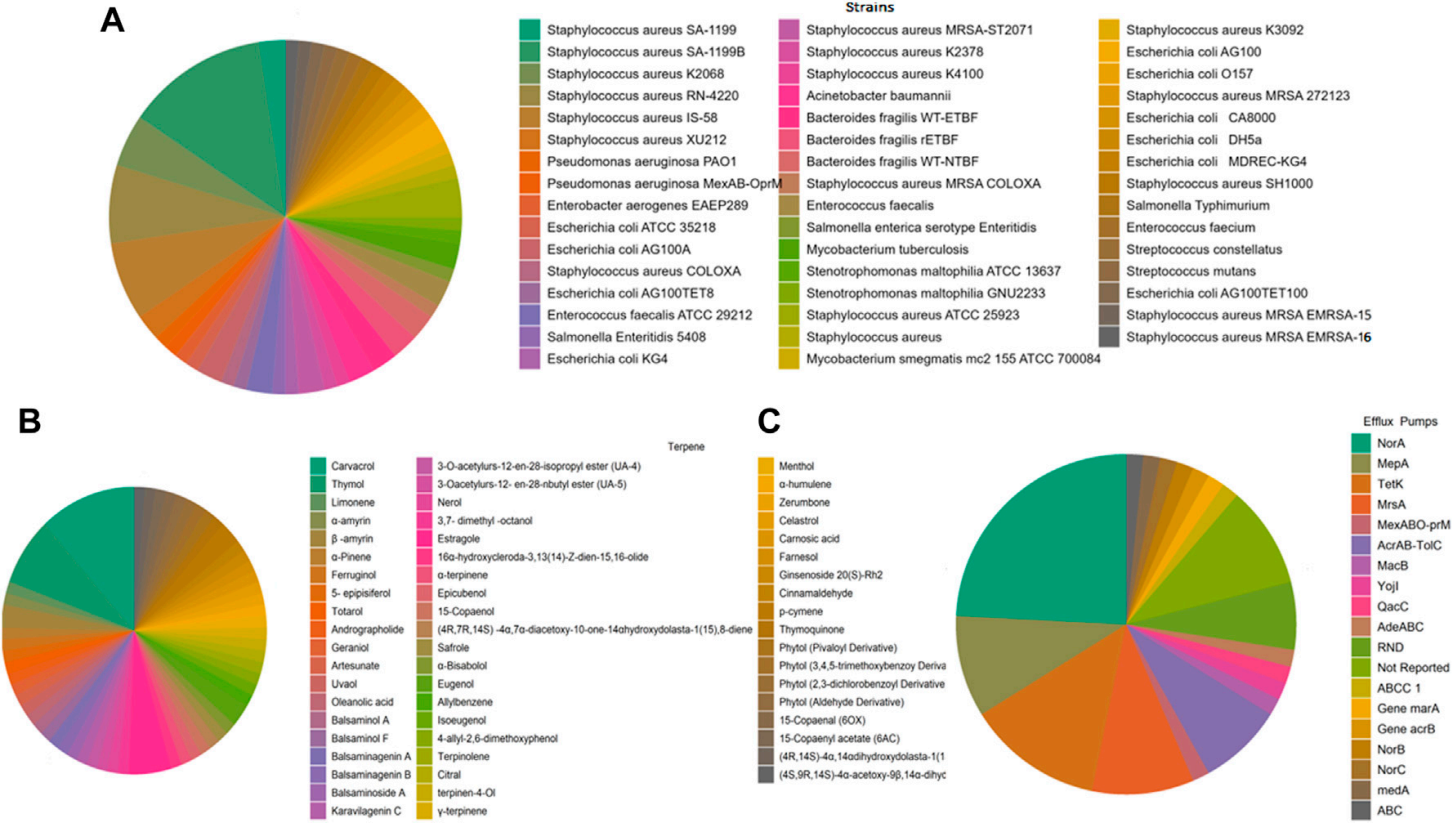
Pie chart plots of frequency analysis. **(A)** Strains; **(B)** terpenes; and **(C)** efflux pumps. These data were analyzed using the Jeffrey Amazing Statistic Program—JASP Team (2022).

**FIGURE 3:**
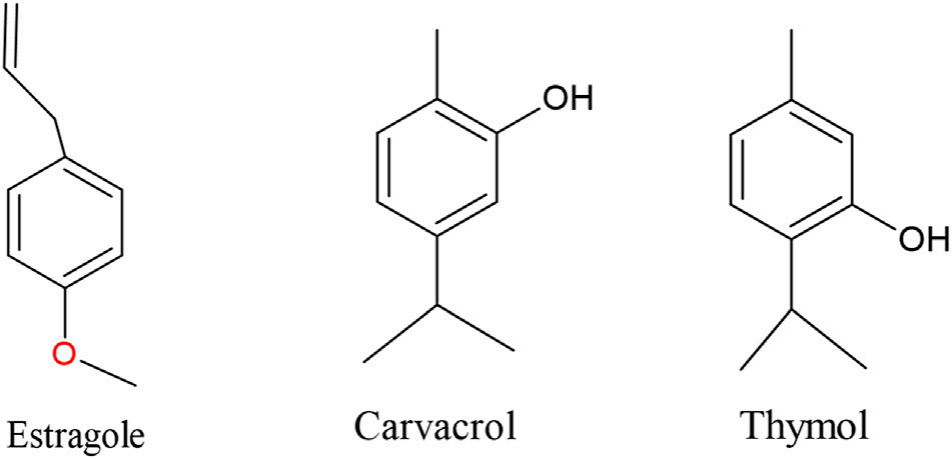
Chemical structures of the most frequently reported terpenes.

In order to guide future research targeting antibacterial drug development, the present review systematically analyzed the inhibitory effects of terpenes against bacterial efflux pumps, reporting the frequency of terpenes, bacterial strains, and efflux pumps (Figure 2). In addition, the results obtained by these studies are described in Table 1. Considering the 41 articles selected for the present review, carvacrol (11,7%), thymol (7.8%), and estragole (5.2%) were the most frequently studied compounds (Figure 2A, Figure 3), demonstrating a prevalence of monoterpenes in the search for natural products as efflux pump inhibitors (the complete analysis of these results is expressed in Supplementary Materials). *Staphylococcus aureus* was the most frequently examined bacterial species in studies of the EPI activities of terpenes (Figure 2B), with emphasis on the following strains: *Staphylococcus aureus* SA-1199B (13.1%), *Staphylococcus aureus* RN-4220 (7.1%), and *Staphylococcus aureus* IS-58 (7.1%) (Supplementary Material).

Concerning the bacterial efflux pumps, the most frequently reported proteins were NorA (24.2%), TetK (12.9%), and MepA (9.7%), corroborating the above-described data on the frequency of bacterial strains. However, it is noteworthy that many studies did not report the type of efflux pump expressed by the tested bacterial strains, which impaired the establishment of a more precise analysis. Most of these studies were published in the last 3 years (Supplementary Material), demonstrating an increasing interest in this topic. It is worth mentioning that a significant number of studies reported the MsrA protein as a target for terpenes. However, strong evidence shows that as a member of the ABC-F family, MsrA proteins are not EP. Instead, they mediate resistance to ribosome targeting antibiotics *via* ribosomal protection (Sharkey et al., 2016; Su et al., 2018; Murina et al., 2019; Wilson et al., 2020). Therefore, although we have maintained these articles in the review, we emphasize the importance of critically interpreting their conclusions.

Following the binary logistic regression analysis, the results showed an association between Gram classification and terpenes (X^2^(gl=1)= 9.13; *p* = 0.03), indicating that these compounds are significantly effective EP inhibitors in both Gram-positive and Gram-negative strains. Notably, the odds ratio (OR) value of 3.03 (95% CI = 1.47–6.23) indicates that Gram-negative bacteria have less chance of being inhibited by a given terpene, i.e., Gram-positive bacteria are probably better targets for further research targeting EP inhibition by terpenes (Table 2).

**TABLE 2:** Binary logistic regression correlating efflux pumps inhibition and the gram classification.

	B	OR	Or (95% CI)	*p*-value
Gram-negative	1.11	3.03	1.47–6.24	0,03*
Gram-positive	1	1	1	**

Note: B: beta; OR: odds ratio; OR (95% CI): Odds Ratio with confidence interval of 95%; *p-value <0.05; ** Category of Reference on the regression: Gram-Positive.

In this study, 75 different terpenes, 63 bacterial strains, and 21 different efflux pumps were reported, with carvacrol, *Staphylococcus aureus* SA-1199B, and NorA appearing as the most frequently mentioned terpene, bacterial strain, and efflux pump, respectively. An analysis of the selected articles revealed that the most common method used to investigate the effectiveness of terpenes as efflux inhibitors is the evaluation of the modification of the Minimum Inhibitory Concentration (MIC) of Ethidium Bromide (EtBr) and Carbonyl Cyanide m-Chlorophenylhydrazine (CCCP). Both these compounds can reduce the efflux of another EP substrate. In this case, a reduction in the MIC EtBr or CCCP resulting from their association with a given terpene indicates that this terpene may be acting as an efflux pump inhibitor. Accordingly, several studies have shown that these terpenes synergize when associated with conventional antibiotics against efflux pump-overexpressing bacterial strains. The MIC determination is the primary method used to analyze *in vitro* the susceptibility to antibiotics and, therefore, is a reliable tool in the evaluation of bacterial resistance, as well as in the identification of potential new therapies (Gunes et al., 2013; Van de Vel et al., 2019; Kowalska-Krochmal and Dudek-Wicher, 2021).

This approach has been adopted to investigate the activity of many compounds reported in this study. Barbosa et al. (2021) investigated the antibacterial activity of thymol and carvacrol against *Staphylococcus aureus* strains 1199 and 1199B, demonstrating that their association with norfloxacin and EtBr resulted in a decreased MIC. Cirino et al. (2014) demonstrated that the same terpenes presented inhibitory effects in association with tetracycline against *S. aureus* strains that overexpress the TetK, NorA, and MsrA proteins; Montagu et al., 2016 showed that both carvacrol and its lipid nanocapsule incorporated formulation presented a synergistic effect with the efflux inhibitor CCCP, indicating interference with the efflux mechanisms of *A. baumannii*. On the other hand, Silveira et al. (2020) found no evidence of EP inhibition in the IS-58 strain of *S aureus* following the association of thymol or carvacrol with EtBr.

Regarding other frequently tested terpenes, a study by Almeida et al. (2020) showed that safrole significantly reduced the MIC of EtBr norfloxacin and ciprofloxacin against the 1199B and K2068 strains of *S. aureus*. Nerol was found to enhance the activity of norfloxacin against the *S. aureus* strain 1199B (Coêlho et al., 2016), while α-terpinene showed synergistic effects with tetracycline and EtBr against the strain IS-58 of the same species (Limaverde et al., 2017). Da costa et al. (2021) showed that the association between estragole and EtBr resulted in synergism against *S. aureus* 1199B and *S. aureus* K2068. On the other hand, Bezerra et al. (2020) verified that the association between estragole and EtBr resulted in antagonism against the strain RN-4220. Using the same bacterial species, Freitas et al. (2021) observed a decrease in the MIC of EtBr and ciprofloxacin following their combination with limonene, while Muniz et al. (2021) reported that eugenol and its derivatives enhanced the effectiveness of norfloxacin and reduced the MIC of EtBr. Additionally, according to Oliveira et al. (2021), the compound α, β-amyrin showed synergistic effects with the efflux inhibitor CCCP against this species. A study by Freitas et al. (2020) demonstrated that α-pinene enhanced the activity of tetracycline against the *Staphylococcus aureus* IS-58 strain, which mainly expresses the TetK EP, suggesting that this compound could be interacting with this protein*.*

*In sílico* modeling was applied by many studies to verify their interaction with these proteins, which was mainly investigated through molecular docking to confirm the interference of monoterpenes with the efflux systems. This approach is commonly used to model the interaction between a small molecule and the binding site of target proteins by predicting the ligand conformation and assessing the binding affinity (Meng et al., 2011). In the previously reported study (Freitas et al., 2020), docking analysis indicated favorable interaction of α-pinene with the active site of the MepA pump. Later, the same group found comparable results when simulating the interaction of limonene and the same protein. Almeida et al. (2020) showed that safrole had favorable interaction with the NorA and MepA efflux pumps, corroborating the *in vitro* results obtained in experiments with *S. aureus* strains. *In silico* analysis by Barbosa et al. (2021) suggested that carvacrol and thymol act as competitive NorA inhibitors, as well as the terpene 4-allyl1-2,6-dimethoxyphenol, investigated by Muniz et al. (2021).

A considerable number of studies reviewed by this work have suggested that terpenes can inhibit the NorA-mediated efflux of antibiotics based mainly on the decrease in antibiotic MIC and consequent synergism resulting from the association of terpenes and conventional antibacterial drugs. Although many of these works have concluded that terpenes act as inhibitors of specific efflux pumps based on their higher expression by the strains investigated, it is essential to note that most of them did not perform molecular tests capable of confirming such conclusions. Therefore, in our opinion, additional tests are needed to ensure that the inhibition of specific efflux pumps corresponds to the mechanism by which terpenes promote synergism when associated with conventional antibiotics or efflux inhibitors such as EtBr and CCCP.

The interference of terpenes in the gene expression of efflux pump components is an important mechanism, in addition to the interference with the activity (as demonstrated in the EtBr test) and interaction with the active site of these proteins (through *in silico* analysis), as demonstrated in several studies included in the present systematic review. In this context, Gupta et al. (2016) demonstrated through Real-Time Quantitative Reverse Transcription PCR (qRT-PCR) that the diterpene clerodane diterpene downregulated the gene expression of efflux pump components, corroborating the findings of Mahmoudi et al. (2020) and Jang and Eom. (2019). On the other hand, Wu et al. (2008) showed that andrographolide alone or combined with standard drugs induced no change in gene expression. The mechanism of action of the monoterpene thymol (Al-Kandari et al., 2019) was associated with the alteration of the membrane permeability and induction of genetic and morphological changes that lead to inhibition of the expression of AcrAB-TolC efflux pump in *Escherichia coli.* Accordingly, Lorenzi et al. (2009) showed that the terpene geraniol inhibited chloramphenicol’s efflux, which was mediated by the AcrAB-TolC system. The inhibition of this efflux system was also observed for ursolic acid against *Escherichia coli* KG4. In addition to inhibiting gene expression of the components of this efflux pump, including AcrAB-TolC, MacB, and Yojl, the terpene was found to reduce the EtBr efflux, as verified through the fluorescence emission intensity method (Dwivedi et al., 2015). This method is based on the principle that EtBr accumulates in cells with low efflux activity, emitting thus higher fluorescence. Of note, this method was used in many studies to analyze terpenes’ interference on different bacterial efflux systems (Jin et al., 2010; Gupta et al., 2017; Freitas et al., 2021); Ramalhete et al., 2011).

In addition to the evidence that terpenes can act as efflux pump inhibitors, it has been demonstrated that these compounds have intrinsic antibacterial activity. Research by Almeida et al. (2020) showed that safrole has significant antibacterial activity against *Staphylococcus aureus* 1199B and K2068, which expresses the NorA and MepA pumps, respectively. Similar findings were obtained by Oliveira-Tintino et al. (2018), who tested the compound α-terpinene against *Staphylococcus aureus* strains 1199 and 1199B. Smith et al. (2007a) and Smith et al. (2007b) analyzed the effectiveness of some terpenes against several strains of *S. aureus* overexpressing different efflux proteins. The authors demonstrated that ferruginol and 5-epipisiferol potentiated the activity of standard antibiotics against these strains, reversing the degree of the observed antibiotic resistance. Moreover, ferruginol inhibited EtBr efflux in the 1199B strain while totarol potentiated the activity of standard antibiotics, in addition to inhibiting the efflux of EtBr in the *Staphylococcus aureus* K3902 strain, which indicates that terpenes may act as inhibitors of different efflux proteins expressed by *S. aureus* strains, such as NorA, MsrA, and TetK. This finding corroborates the work of Cruz et al. (2020). They demonstrated that the antibacterial activity of the sesquiterpene α-bisabolol against the IS-58 strain of *Staphylococcus aureus* is associated with the inhibition of the TetK efflux pump.

Finally, it is essential to emphasize that terpenes can also function as structural models for the obtention of derivatives with improved pharmacological properties, which may significantly contribute to the discovery of new antibacterial drugs (Daouda et al., 2014; Melo-Coutinho et al., 2015).

## Conclusion

Terpenes are compounds with significant antibacterial activity against both Gram-positive and Gram-negative strains. While some compounds showed no clinically relevant intrinsic antibacterial effects, their association with conventional antibiotics frequently resulted in synergistic effects, indicating enhanced antibiotic activity.

**FIGURE 4:**
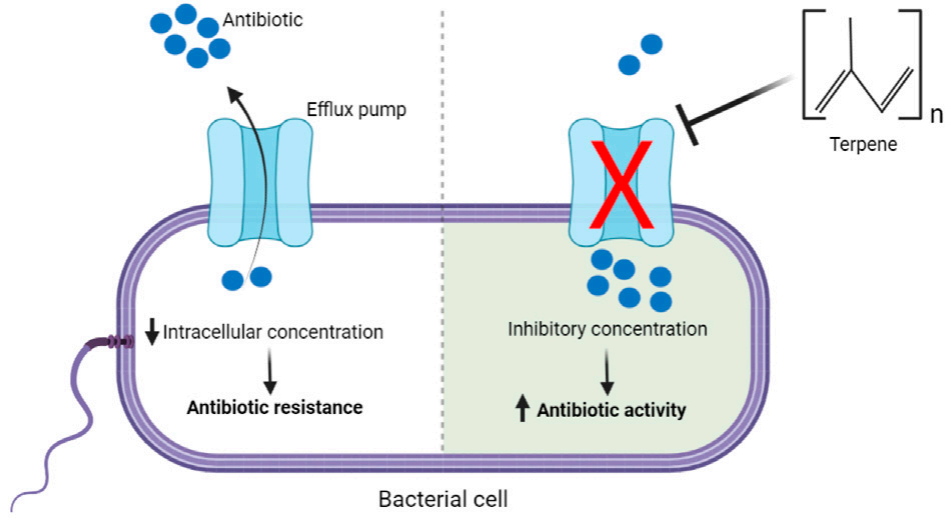
Illustration of the inhibition of bacterial efflux pumps by terpenes and its impact on antibiotic resistance.

The investigation of terpenes as efflux pump inhibitors in *S. aureus* strains is the most analyzed, with NorA and carvacrol being the most investigated terpene and efflux pump, respectively. Importantly, Efflux pumps of Gram-positive bacterial strains are probably more susceptible to the action of terpenes, which involve either gene expression inhibition or the interaction with the binding site of membrane-associated efflux proteins, although the molecular mechanisms underlying the action of terpenes as EP inhibitors remain to be better elucidated (Figure 4).

In conclusion, terpenes are significant efflux pump inhibitors in a wide variety of bacterial strains and can potentially be used in drug development to combat antibiotic resistance.

## Data Availability

The original contributions presented in the study are included in the article/Supplementary Material, further inquiries can be directed to the corresponding author.
