# Supplementary material for: Terpenes as bacterial efflux pump inhibitors: A systematic review
Source: Front Pharmacol. 2022 Oct 13;13:953982. doi: 10.3389/fphar.2022.953982 (PMC9606600; doi:10.3389/fphar.2022.953982)
Supplement: Supplementary file 1 [file DataSheet1.PDF]

Supplementary Materials

Supplementary Table 1. Frequencies of the Publication Years

Year	N	Percent
2021	4	9.756
2020	9	21.951
2019	5	12.195
2018	2	4.878
2017	3	7.317
2016	3	7.317
2015	1	2.439
2014	4	9.756
2013	1	2.439
2012	1	2.439
2011	3	7.317
2010	1	2.439
2009	1	2.439
2008	1	2.439
2007	2	4.878
Total	41	100.000

Supplementary Table 2. Frequencies of the Terpenes

**Terpene**	**N**	**Percent**
Carvacrol	9	11.688
Thymol	6	7.79
Limonene	1	1.299
α-amyrin	1	1.299
β -amyrin	2	2.597
α-Pinene	1	1.299
Ferruginol	1	1.299
5-epipisiferol	1	1.299
Totarol	1	1.299
Andrographolide	1	1.299
Geraniol	1	1.299
Artesunate	1	1.299
Uvaol	1	1.299
Oleanolic acid	1	1.299
Balsaminol A	1	1.299
Balsaminol F	1	1.299
Balsaminagenin A	1	1.299

Balsaminagenin B	1	1.299
Balsaminoside A	1	1.299
Karavilagenin C	1	1.299
3-O-acetylurs-12-en-28-isopropyl ester (UA-4)	1	1.299
3-Oacetylurs-12- en-28-nbutyl ester (UA-5)	1	1.299
Nerol	1	1.299
3,7-dimethyl -octanol	1	1.299
Estragole	4	5.195
16α-hydroxycleroda-3,13(14)-Z-dien-15,16-olide	1	1.299
α-terpinene	1	1.299
Epicubenol	1	1.299
15-Copaenol	1	1.299
(4R,7R,14S)-4α,7α-diacetoxy-10-one-14αhydroxyd	1	1.299

olasta-1(15),8-diene		
Safrole	1	1.299
α-Bisabolol	1	1.299
Eugenol	2	2.597
Allylbenzene	1	1.299
Isoeugenol	1	1.299
4-allyl-2,6-dimethoxyphenol	1	1.299
Terpinolene	1	1.299
Citral	1	1.299
terpinen-4-Ol	1	1.299
γ-terpinene	1	1.299
Menthol	1	1.299
α-humulene	1	1.299
Zerumbone	1	1.299
Celastrol	1	1.299
Carnosic acid	1	1.299
Farnesol	1	1.299
Ginsenoside 20(S)-Rh2	1	1.299

Cinnamaldehyde	1	1.299
p-cymene	2	2.597
Thymoquinone	1	1.299
Phytol (Pivaloyl Derivative)	1	1.299
Phytol (3,4,5-trimethoxybenzoy Derivative)	1	1.299
Phytol (2,3-dichlorobenzoyl Derivative)	1	1.299
Phytol (Aldehyde Derivative)	1	1.299
15-Copaenal (6OX)	1	1.299
15-Copaenyl acetate (6AC)	1	1.299
(4R,14S)-4α,14αdihydroxydolasta-1(15),8-diene	1	1.299
(4S,9R,14S)-4α-acetoxy-9β,14α-dihydroxydolasta-	1	1.299

1(15),7-diene		
Total	77	100.000

Supplementary Table 3. Frequencies for Strains

Bacteria	Frequency	Percent
Staphylococcus aureus SA-1199	2	2.381
Staphylococcus aureus SA-1199B	11	13.095
Staphylococcus aureus K2068	4	4.762
Staphylococcus aureus RN-4220	6	7.143
Staphylococcus aureus IS-58	6	7.143
Staphylococcus aureus XU212	2	2.381
Pseudomonas aeruginosa PAO1	1	1.190

Pseudomonas aeruginosa MexAB-OprM	1	1.190
Enterobacter aerogenes EAEP289	1	1.190
Escherichia coli ATCC 35218	1	1.190
Escherichia coli AG100A	2	2.381
Staphylococcus aureus COLOXA	1	1.190
Escherichia coli AG100TET8	1	1.190
Enterococcus faecalis ATCC 29212	2	2.381
Salmonella Enteritidis 5408	1	1.190
Escherichia coli KG4	1	1.190
Staphylococcus aureus MRSA-ST2071	2	2.381

Staphylococcus aureus K2378	1	1.190
Staphylococcus aureus K4100	1	1.190
Acinetobacter baumannii	2	2.381
Bacteroides fragilis WT-ETBF	2	2.381
Bacteroides fragilis rETBF	2	2.381
Bacteroides fragilis WT-NTBF	2	2.381
Staphylococcus aureus MRSA COLOXA	1	1.190
Enterococcus faecalis	2	2.381
Salmonella enterica serotype Enteritidis	1	1.190
Mycobacterium tuberculosis	2	2.381
Stenotrophomonas maltophili	1	1.190

a ATCC 13637		
Stenotrophomonas maltophilia GNU2233	1	1.190
Staphylococcus aureus ATCC 25923	3	3.571
Staphylococcus aureus	1	1.190
Mycobacterium smegmatis mc2 155 ATCC 700084	1	1.190
Staphylococcus aureus K3092	1	1.190
Escherichia coli AG100	2	2.381
Escherichia coli O157	1	1.190
Staphylococcus aureus MRSA 272123	1	1.190
Escherichia coli CA8000	1	1.190

Escherichia coli DH5a	1	1.190
Escherichia coli MDREC-KG4	1	1.190
Staphylococcus aureus SH1000	1	1.190
Salmonella Typhimurium	1	1.190
Enterococcus faecium	1	1.190
Streptococcus constellatus	1	1.190
Streptococcus mutans	1	1.190
Escherichia coli AG100TET100	1	1.190
Staphylococcus aureus MRSA EMRSA-15	1	1.190
Staphylococcus aureus MRSA EMRSA-16	1	1.190

Missing	0	0.000
Total	84	100.000

Supplementary Table 4. Frequencies of the Efflux Pumps

**Efflux_Pumps**	**N**	**Percent**
NorA	15	24.194
MepA	6	9.677
TetK	8	12.903
MrsA	6	9.677
MexABO-prM	1	1.613
AcrAB-TolC	5	8.065
MacB	1	1.613
YojI	1	1.613
QacC	1	1.613
AdeABC	1	1.613
RND	4	6.452
Not Reported	6	9.677
ABCC 1	1	1.613
Gene marA	1	1.613

Gene acrB	1	1.613
NorB	1	1.613
NorC	1	1.613
medA	1	1.613
ABC	1	1.613
Total	62	100.000
